# Epigenetic landscape of 5-hydroxymethylcytosine and associations with gene expression in placenta

**DOI:** 10.1080/15592294.2024.2326869

**Published:** 2024-03-20

**Authors:** Michael Mortillo, Elizabeth G. Kennedy, Karen M. Hermetz, Amber A. Burt, Carmen J. Marsit

**Affiliations:** Gangarosa Department of Environmental Health, Rollins School of Public Health, Emory University, Atlanta, GA, USA

**Keywords:** Hydroxymethylation, gene expression, eQTHM, placenta

## Abstract

5-hydroxymethylcystosine (5hmC), is an intermediate product in the DNA demethylation pathway, but may act as a functional epigenetic modification. We have conducted the largest study of site-specific 5hmC in placenta to date using parallel bisulphite and oxidative bisulphite modification with array-based assessment. Incorporating parallel RNA-sequencing data allowed us to assess associations between 5hmC and gene expression, using expression quantitative trait hydroxymethylation (eQTHM) analysis. We identified ~ 47,000 loci with consistently elevated (systematic) 5hmC proportions. Systematic 5hmC was significantly depleted (*p* < 0.0001) at CpG islands (CGI), and enriched (*p* < 0.0001) in ‘open sea’ regions (CpG >4 kb from CGI). 5hmC was most and least abundant at CpGs in enhancers and active transcription start sites (TSS), respectively (*p* < 0.05). We identified 499 significant (empirical-p <0.05) eQTHMs within 1 MB of the assayed gene. At most (75.4%) eQTHMs, the proportion of 5hmC was positively correlated with transcript abundance. eQTHMs were significantly enriched among enhancer CpGs and depleted among CpGs in active TSS (*p* < 0.05 for both). Finally, we identified 107 differentially hydroxymethylated regions (DHMRs, *p* < 0.05) across 100 genes. Our study provides insight into placental distribution of 5hmC, and sheds light on the functional capacity of this epigenetic modification in placenta.

## Introduction

The placenta is an organ that acts as the interface between the foetus and its mother [1]. Its crucial roles include nutrient transfer, gas exchange, waste removal, immune protection, and various neuroendocrine functions [2–4]. Disruptions in placental processes can lead to pregnancy complications including preeclampsia, inflammation, preterm delivery and foetal growth restriction, with subsequent implications for the health of both the infant and the mother [5–7].

5-methylcytosine (5mC) is formed through DNA methyltransferases (DNMTs) adding a methyl group to the 5^th^ carbon position of an unmethylated cytosine [8]. It is one of the most abundant DNA modifications in the human genome and is involved in a number of epigenetic functions, including modulation of transcription factor binding to regulatory regions [9]. When found in the promoter regions of genes, it generally leads to repression in transcription [10]. 5-hydroxymethylcytosine (5hmC) is produced through enzymatic oxidation of 5mC by ten-eleven translocation (TET) methylcytosine dioxygenases [11,12]. Though it is most commonly believed that 5hmC acts as an intermediary in the DNA demethylation pathway, there is also evidence to suggest that 5hmC is stable and could act as a stand-alone epigenetic modification [13]. Within the gene body, 5hmC is correlated with increased transcription, depending on cell and tissue type [14,15]. In the placenta, 5hmC is sparse [16], and has been observed to be enriched at imprinted loci [17], although when evaluated by Piyasena et al. [18] at imprinting control regions (ICRs) of the genes IGF2, H19, and CDKN1C, was not associated with gene expression. Our group has previously leveraged publicly available placental gene expression data to demonstrate that 5hmC is positively associated with transcription in actively transcribed genes, but did not assess 5hmC and gene expression in the same samples [19].

In this study, we aim to characterize 5hmC distribution across the placenta and identify specific areas of 5hmC that relate to gene expression through the use of expression quantitative trait hydroxymethylation (eQTHM). We believe that drawing a direction correlation between 5hmC and expression will shed light on the functionality and distribution of placental 5hmC and provide a framework for future studies of the placental epigenome.

## Materials and methods

### Study population

The study was conducted in placenta samples collected from participants enrolled in the Rhode Island Child Health Study (RICHS). This hospital-based cohort (*n* = 840) recruited women 18–40 years old, with no history of psychological disorders and in good physical health, and who delivered infants from healthy, non-pathologic pregnancies at term (≥37 gestational weeks). Mother–infant pairs were recruited between 1 September 2009 and 31 July 2014 from the Women and Infants Hospital of Rhode Island (WIH, Providence, RI). The cohort was established to examine the relationship between molecular features of the placenta and birthweight and was oversampled for infants born small for gestational age (SGA, <10% 2013 Fenton Growth Curve) and large for gestational age (LGA, >90% 2013 Fenton Growth Curve), each matched on gender, gestational age, and maternal age to infants born appropriate for gestational age (AGA, 10–90% 2013 Fenton Growth Curve) [20].

Participant demographic data was collected from interviewer-administered questionnaires and clinical data were abstracted from a structured review of medical records. The current study focuses on a subset of the enrolled participants with available placental 5hmC data (*n* = 227).

### Placental sample collection

Placental parenchyma was obtained within 2 hours of delivery, taken from the foetal side of the placenta, 2 cm from the umbilical cord insertion site. All samples were free of maternal decidua. Samples were placed in RNALater at 4°C. At least 72 hours later, samples were removed from RNALater, snap-frozen in liquid nitrogen, pulverized to homogenize the samples, and stored at −80°C until extraction.

### CpG methylation and hydroxymethylation profiling

Hydroxymethylation profiling was performed as previously described [19]. Briefly, DNA was extracted using the DNeasy Blood and Tissue Kit (Qiagen, Germantown, MD, USA) following manufacturer’s protocol, quantified with the Qubit Fluorometer (Thermo Fisher Scientific Life Sciences), and stored at −80°C. Bisulphite (BS) and oxidative bisulphite (oxBS) conversion were performed on placenta-derived DNA using the TrueMethyl oxBS Module (NuGen, Redwood City, CA, USA), following manufacturer’s optimized protocol of 500 ng gDNA input for downstream analysis using the Infinium HD Methylation EPIC Bead Chip Array (Illumina, San Diego, CA, USA).

Cross-reactive CpG probes [21], probes that failed detection p-value (*p* > 0.01) in >1 sample, and probes overlapping single nucleotide polymorphisms (SNPs) were removed from the analysis. Three samples were removed due to failing sex quality control or failing detection p-value (p-value >0.01 in >2% of probes). After quality control, 706,435 CpGs were available for normalization. Normalization of background correction and dye bias of raw signals from each of the BS and oxBS-converted samples was done using the Noob procedure, followed by normalization of probe-type bias using SWAN, both of which are available in the R/Bioconductor package minfi (version 1.24.0; *https://www.bioconductor.org*). Estimation of 5hmC beta values (proportion of CpGs across all cells in a given sample that are hydroxymethylated) for each CpG on the array was performed using OxyBS (version 1.5) [22]. Potential confounding due to array chip was removed using the ComBat function in R/bioconductor package sva [23]. Beta values were logit-transformed (M-values) to better approximate a normal distribution [24]. Finally, we limited our analysis to autosomal probes only, to avoid any confounding due to sex-specific effects. The final filtered, normalized dataset contained 689,815 CpGs.

### RNA-sequencing, quality control and read filtering

RNA-sequencing was performed as previously described [25]. Briefly, Total RNA was isolated from homogenized placental tissue, stored, and quantified. RNA was then converted to cDNA, and transcriptome-wide 50 bp single-end RNA sequencing was conducted using the HiSeq 2500 platform (Illumina, San Diego, CA) [26]. Samples were run in three sequencing batches, with 10% of the samples run in triplicate within each batch.

The raw RNA sequencing data (fastq files) were assessed for quality control, including read length and GC content. Reads that passed quality control were mapped to the human reference genome (hg19) in a splice-aware manner, with common SNPs in the reference genome masked prior to alignment. Genes with counts per million <1 in greater than 30 samples were considered unexpressed and removed. Read counts were adjusted for GC content, followed by TMM correction [27] for library size differences across samples. The data were then log_2_-transformed to account for the mean–variance relationship. Following assessments of Pearson correlations in gene expression among the triplicate samples, duplicated repeat samples were removed from the analysis. The data were adjusted to remove batch effect due to flow cell, using the ComBat function in R/bioconductor package sva [23]. We then removed poorly defined transcripts (transcripts containing the phrases ‘LOC,’ ‘orf,’ ‘KIAA,’ ‘NCRNA,’ and ‘MIR’), as well as genes on sex chromosomes. The final filtered, normalized dataset contained reads mapped to 12,744 genes. For our 5hmC-expression analysis (see section below, ‘*eQTHM Identification*’), we used samples that had both placental 5hmC *and* gene expression data available (*n* = 197). To assess 5hmC enrichment across genes with varying expression levels, we grouped our genes into expression quartiles (0–25%, 26% −50%, 51% −75%, 76% −100%) based on mean log_2_TMM expression counts across all samples.

### Annotation

We annotated CpGs using the R/bioconductor package ‘IlluminaHumanMethylationEPICanno.ilm10b2.hg19’ [28]. This includes annotating each CpG based on its location relative to the nearest gene, as well as annotating CpGs that fall within a CpG islands (CGI) interval. The available gene compartments from the EPIC array package are as follows: 1) 5’ untranslated region (5’ UTR), 2) TSS 200 (1–200 base pairs (bp) upstream of the TSS), 3) TSS 1500 (201–1500 bp upstream of TSS), 4) 1^st^ exon, 5) gene bodies, 6) exon boundaries, and 7) 3’ untranslated region (3’ UTR). CpGs that fell in either the TSS200 or TSS1500 intervals were combined into one ‘TSS’ interval. CpGs that fell into gene bodies, 1^st^ exons, or exon boundaries were combined into one ‘gene body’ class. For the CGI regions, CpGs were annotated to a CGI ‘shore’ if they were within a 2 kb region flanking a CGI, a CGI ‘shelf’ if they were within a 2 kb region flanking a CGI shore, or an ‘open sea’ if they were not within a shore, shelf, or CGI. CpGs were also annotated to chromatin-based genomic categories using ChromHMM [29], derived from the Roadmap Epigenomics Consortium [30] and applied to foetal placenta cells. We combined enhancers and genic enhancers into one ‘enhancers’ class, strong and weak transcription into a ‘transcribed’ class, bivalent enhancers and bivalent/poised transcription start sites (TSS) into ‘poised TSS/enhancers’ class, and finally polycomb repressed and weak polycomb repressed into a single ‘polycomb repressed’ class.

In order to relate the location of each CpG to its associated gene for eQTHM analysis, gene TSS and transcription termination site (TTS) positions were obtained from the switchDbTss track of the UCSC table browser (hg19/GRCh37) [31]. 5’ and 3’UTR positions were obtained from the NCBI RefSeq track of the table browser. When multiple TSS, TTS, 5’UTR, and 3’UTR positions were annotated for one gene, we used the most 5’ upstream and most 3’ downstream position. To annotate gene body coordinates, we selected the most 5’ upstream start position of the 1^st^ exon and the most 3’ downstream end position of the last exon.

When mapping the distance of the CpG to its target eQTHM gene, CpGs upstream of the TSS were calculated as negative bp to the TSS, while CpGs downstream of the TTS were calculated as positive bp. For CpGs that fall within their target gene, their distance is represented as the proportion of the way through the gene (to account for variability in gene length).

### Systematic 5hmC distribution

As in prior work [19], CpGs at which 5hmC proportion ≥0.10 in at least 50% of samples were defined as regions of systematic 5hmC (*n* = 113). Distribution of systematic 5hmC in relation to nearest genes and CGI feature was assessed using odds ratios (ORs) and 95% confidence intervals (CIs) derived from a Fisher’s exact test. This allowed us to compare the proportions of loci demonstrating systematic 5hmC within a gene/CGI compartment against loci not demonstrating systematic 5hmC.

### eQTHM identification

eQTHM is an extension of expression quantitative trait methylation (eQTM), a method used to identify specific positions in the genome where the proportion of methylated CpGs at one locus is associated with transcript abundance for a given gene [32]. In the current study, we assess how 5hmC proportion at a given CpG in the placental epigenome associates with the abundance of a gene transcript. To this end, we have conducted a cis eQTHM analysis using the Matrix eQTL R package [33]. Matrix eQTL fits a multivariate linear regression model: y = β_0_ + β_1_HM + Tα + ε, where y is the normalized transcript counts for each gene, HM is 5hmC M-values at each CpG, β_0_, β_1_, and α are regression coefficients, and T represents covariates. We identified covariates as any variable with significant univariate association (*p* < 0.05) with any of the top five principal components (PCs) for the 5hmC data and any of the top five PCs of the transcriptomic data. From this, we identified infant sex, birthweight percentile, and estimated proportions of syncytiotrophoblasts (STBs, estimated using R/Bioconductor package planet [34]) as being significantly associated with both pairs of PCs, and included them as covariates in our model. With respect to the transcript under investigation, we restricted our analyses to only CpGs that were 1) in the gene, 2) up to 1 Mb upstream of the transcription start site (TSS), and 3) up to 1 Mb downstream of the transcription termination site (TTS). We then implemented an empirical p-value approach (see section below) to find eQTHMs among all cis CpGs. Among eQTHMs, we conducted a Pearson’s correlation analysis between 5hmC at the CpG and transcript level of the target gene, according to the Matrix eQTL guidelines [33].

#### Empirical p-value threshold

CpGs can be mapped to multiple genes that fall within a 1 Mb window, so we employed an empirical p-value threshold to ensure that genes paired to a higher number of CpGs do not have a greater chance of being part of an eQTHM. We defined a significance threshold using the procedure previously described for eQTL analyses in the Genotype-Tissue Expression (GTEx) project [35], and subsequently for expression quantitative trait methylation (eQTM) analysis by Ruiz-Arenas et al. [32] Briefly, we performed the eQTHM analysis described above on data with shuffled sample identifiers, collecting the minimum p-value obtained for each gene in the permutation. We obtained 1,000 permuted p-values for each gene. We modelled the null distribution with the permuted *P*-values using a beta distribution, generating the parameters with maximum likelihood estimation [36]. Using the beta distributions, we estimated empirical p-values for the minimum observed (non-permuted) p-values obtained for each gene. False discovery rates (FDR) were calculated for gene-wise empirical p-values using the Benjamini–Hochberg method [37]. Genes with FDR less than 5% were considered significant. Finally, we defined an empirical p-value threshold as the empirical p-value of the gene closest to the 5% FDR threshold (i.e., the highest empirical p-value among genes significant at 5% FDR). Among all cis CpG-gene pair associations, if the nominal p-value for the CpG was less than the empirical threshold *and* the corresponding gene had an FDR < 0.05, it was deemed an eQTHM.

### Differentially hydroxymethylated regions (DHMRs)

We also conducted a DHMR analysis, where we aimed to find contiguous local genomic regions with differential hydroxymethylation associated with gene expression, considering these regions may be more biologically relevant than individual eQTHMs. To perform this, we utilized the Comb-p method [38], which identifies regions enriched for low p-values (using Matrix eQTL p-values). Comb-p corrects p-values for auto-correlation with neighbouring CpGs (within 1 kb) using the Stouffer–Liptak method, and then builds DHMRs for a specific gene based on CpGs with corrected p-values falling below a threshold. We specified a corrected p-value threshold of 1e-4. Once a DHMR is identified, a regional p-value is generated and adjusted for multiple testing using the Sidak correction. DHMRs with an adjusted regional p-value <0.05, *and* containing at least three CpGs were considered significant. This process was conducted for every gene in our eQTHM analysis.

### Enrichment tests

Fisher’s exact tests were used to test for enrichment of systematic 5hmC within gene compartments and CGI regions, as well as enrichment of systematic CpGs among CpGs in the eQTHMs and DHMRs. Fisher’s exact tests were also used to test for enrichment of positively and negatively correlated eQTHMs in target gene compartments, CGI regions, and ChromHMM states, as well as enrichment of DHMR CpGs across ChromHMM states. One-way repeated-measures analysis of variance (ANOVA) was used to test for differences in 5hmC proportions within gene compartments of genes with varying expression levels, as well as to assess 5hmC differences across ChromHMM compartments.

All analyses were conducted using R version 4.1.1. See Figure S1 for workflow of main analyses.

## Results

### Sample cohort

This study analysed data from 227 mother-infant pairs from the RICHS cohort, with the mother and infant demographics displayed in Table 1. The sample consisted mainly of white mothers (77.1% of samples), with a mean age of 30.9 years. There was a nearly equal distribution of male and female infants (51.1% vs. 48.9%, respectively), with a mean gestational age of 39.4 weeks, and by study design, the sample was over-represented by infants born SGA (14.5%) and LGA (30.8%).

**Table 1.** RICHS participant demographics.

	RICHS (*n* = 227)
**Maternal characteristics**	
Age in years (mean, SD)	30.9 (4.9)
Educational attainment (n, %)	
High school or less	35 (15.4)
Post-high school or junior college	111 (48.9)
College	81 (35.7)
Self-reported race/ethnicity (n, %)	
Asian	10 (4.4)
Black	12 (5.3)
Indian	2 (0.88)
More than one race	4 (1.8)
Unknown/not reported	24 (10.6)
White	175 (77.1)
**Infant characteristics**	
Age in weeks (mean, SD)	39.4 (0.9)
Sex (n, %)	
Male	116 (51.1)
Female	111 (48.9)
Birthweight in grams (mean, SD)	3,556.4 (662.5)
Birthweight category (n, %)^a^	
AGA	124 (54.6)
LGA	70 (30.8)
SGA	33 (14.5)

^a^Infants born with birthweight percentile ≤ 10% (small for gestational age [SGA]), 10–90% (appropriate for gestational age [AGA]), and ≥ 90% (large for gestational age [LGA])

### Placental 5hmC distribution

**Figure 1.**
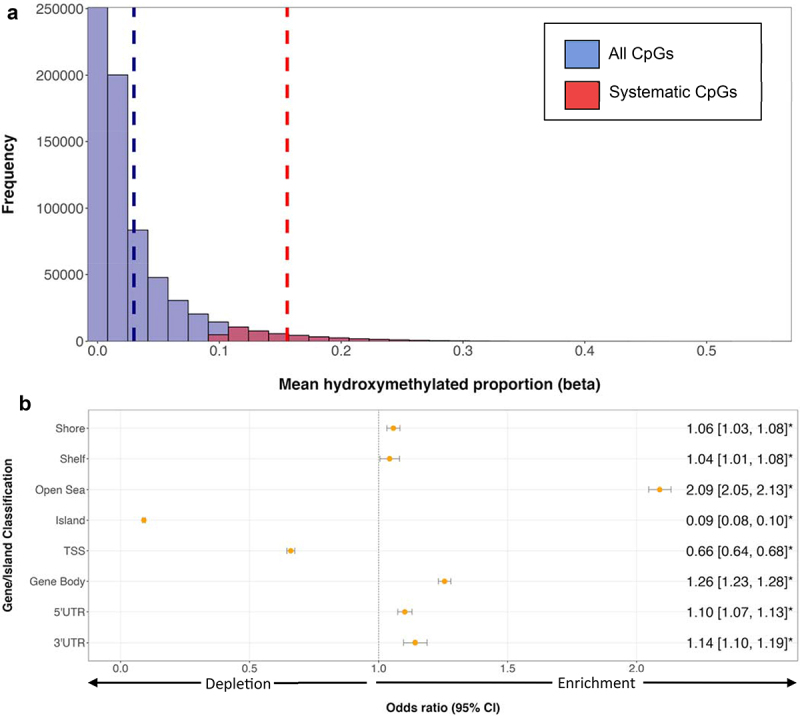
Distribution of systematic 5hmC across the placental epigenome. Systematic CpGs were defined as loci with 5hmC proportion > 0.10 in at least 50% (*n* = 113) of samples. A total of 689,815 autosomal CpGs were assayed. Among those 46,921 (6.8%) were considered systematic, with the remaining 642,894 (93.2%) deemed non-systematic. (a) distribution of all CpGs sites (blue) and systematic sites (red) on EPIC array. 5hmC proportions display a strong right skew, with samples having a mean of 2.98% (indicated by the vertical dashed blue line). Systematic CpGs had a 5hmC mean of 15.57% across samples (indicated by vertical dashed red line). (b) distribution of systematic 5hmC by gene and CGI compartments. ORs and 95% CIs were determined by Fisher’s exact test, with ORs marked by asterisks defined as significant (*p* < 0.05). ORs above 1.0 indicate enrichment for systematic 5hmC in comparison to other location classifiers, and ORs below 1.0 indicate depletion. CpGs associated with > 1 gene class may be counted twice. CGI shores define loci < 2 kb from CGI, shelves are loci 2–4 kb from CGI, and open seas are loci > 4 kb from CGI.

Placental 5hmC proportions were notably low with very little variation. Mean 5hmC across all 689,815 autosomal CpGs ranged from 0% to 56%, with the 227 samples having a grand mean of 2.98% (Figure 1(a)). Among all CpGs 46,921 (6.8%) were deemed systematic (5hmC proportion ≥0.10 in at least 50% of samples), with a mean 5hmC of 15.57% (Figure 1(a)).

Across gene compartments, we observed significant enrichment of systematic 5hmC in gene bodies (OR = 1.26; 95% CI = 1.23, 1.28; *p* < 0.0001), 3’ UTRs (OR = 1.14; 95% CI = 1.10, 1.19; *p* < 0.0001), and 5’ UTRs (OR = 1.10; 95% CI = 1.07, 1.13; *p* < 0.0001). Significant depletion of systematic 5hmC was observed in TSS (OR = 0.66; 95% CI = 0.64, 0.68; *p* < 0.0001) (Figure 1(b)). Among CGI regions, we observed a significant enrichment of systematic 5hmC at ‘open sea’ regions (OR = 2.09; 95% CI = 2.05, 2.13; *p* < 0.0001), and depletion in CGIs (OR = 0.09; 95% CI = 0.08, 0.10; *p* < 0.0001) (Figure 1(b)).

**Figure 2.**
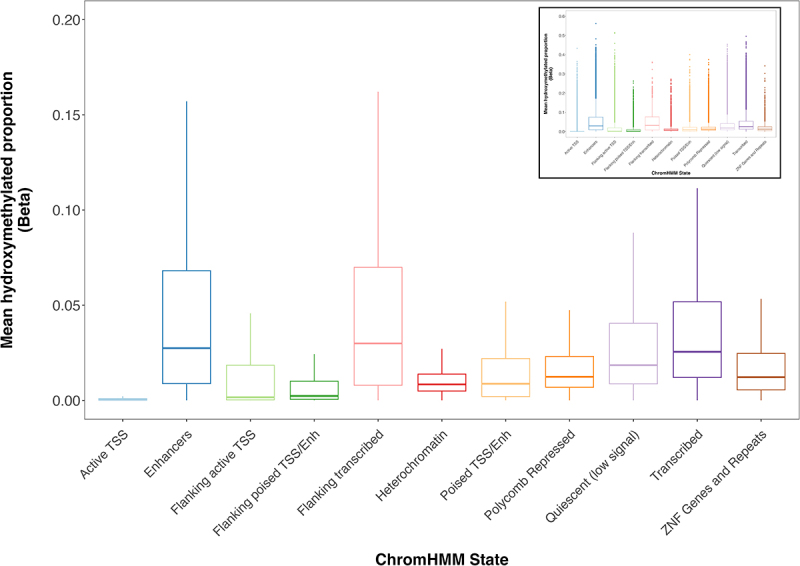
Placental 5hmC distribution across ChromHMM states. Box plots denote distribution of 5hmC, with boxes encompassing 25th to 75th percentile (with the length of the box representing the interquartile range (IQR), defined as the difference between the 25th and 75th percentiles), the median denoted as line within box, and the upper and lower whiskers marking the maximum and minimum values no further than 1.5 x IQR, respectively. Outliers were suppressed to improve visualization of differences. Inset plot represents the plot with outliers included. One-way repeated measures ANOVA revealed significant differences in 5hmC levels across states (*p* < 0.05).

Across ChromHMM states, mean 5hmC was most abundant among enhancer CpGs (mean = 5.2%), flanking transcribed (mean = 5.1%), and transcribed regions (mean = 4.2%). 5hmC was least abundant among CpGs found in active TSS, flanking poised TSS/enhancers, and flanking active TSS (means = 0.39%, 1.1%, and 2.0%, respectively) (Figure 2). Significant differences in 5hmC proportions were found across all ChromHMM states (*p* < 0.05).

**Figure 3.**
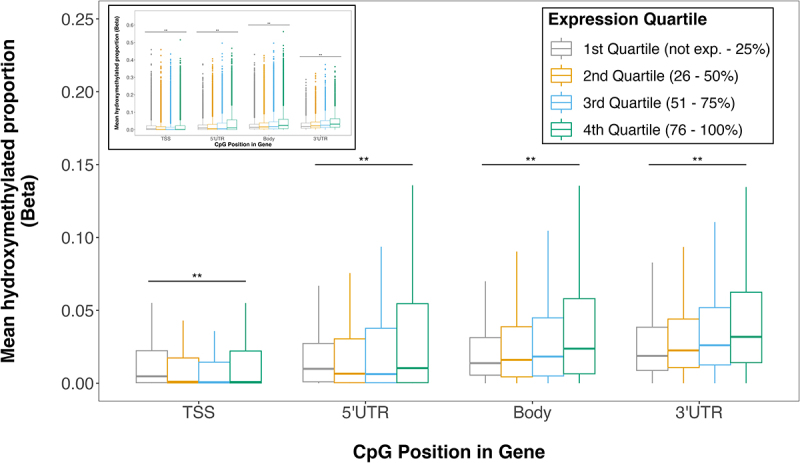
Placental 5hmC distribution across gene compartments of genes with varying expression levels. Genes were grouped into expression quartiles based on mean transcript levels across all subjects. CpG probes were mapped to compartment of nearest gene from EPIC array annotation package. Box plots denote distribution of 5hmC, with boxes encompassing 25^th^ to 75^th^ percentile (with the length of the box representing the IQR, defined as the difference between the 25^th^ and 75^th^ percentiles), the median denoted as line within box, and the upper and lower whiskers marking the maximum and minimum values no further than 1.5 × IQR, respectively. Outliers were suppressed to improve visualization of differences. Inset plot represents the plot with outliers included. Asterisks mark significant differences in 5hmC levels across expression quartiles within each gene compartment (ANOVA *p* < 0.05).

We found that 5hmC proportions varied by both genic region and gene expression level. Although proportions of 5hmC were largely dependent on the location of the CpG site relative to the gene, hydroxymethylation proportion generally increased with transcript abundance. 5hmC among CpGs lying in the gene body or 3’ UTR increased as transcript abundance increased from transcriptionally silent (represented by the first quartile of transcript abundances) to active genes (fourth quartile) (Figure 3). Among CpGs in the 5’ UTR, 5hmC proportion was highest in transcriptionally silent genes, then decreased as transcription increased, and finally increased slightly in the most actively transcribed genes. 5hmC was negatively correlated with transcript in TSS; as transcript abundance increased, 5hmC proportion decreased. Within all gene compartments, 5hmC proportion differed across expression quartiles (*p* < 0.05, for all) (Figure 3). Thus, we observed significant differences in 5hmC proportions across expression quartiles, indicating a potential association between 5hmC and expression.

### eQTHM analysis

We identified 499 eQTHMs (Table S1), consisting of 473 unique CpGs and 284 unique genes. Among the 473 unique CpGs, 165 (34.9%) were deemed systematic CpGs from our placental 5hmC distribution results, representing a significant enrichment (OR = 6.79; 95% CI = 5.60, 8.21; *p* < 0.0001). We also observed an overall positive correlation between 5hmC and expression, with 75.4% of significant eQTHMs demonstrating a positive correlation with cis gene transcript abundance (Table 2).

**Table 2.** eQTHM summary results.

	All cis CpG-gene pairs (*n* = 19,546,660)
Pairs below empirical-p threshold (n, %)	499 (0.003)
# unique CpGs	473
# unique genes	284
Unique CpGs that overlap with systematic CpGs (n, %)	165 (34.9)
Correlation sign (n, %)	
+	376 (75.4)
-	123 (24.6)
Estimate (mean, SD)	0.29 (0.46)
r (mean, SD)	0.20 (0.36)

Abbreviations: *r* = correlation coefficient.

**Figure 4.**
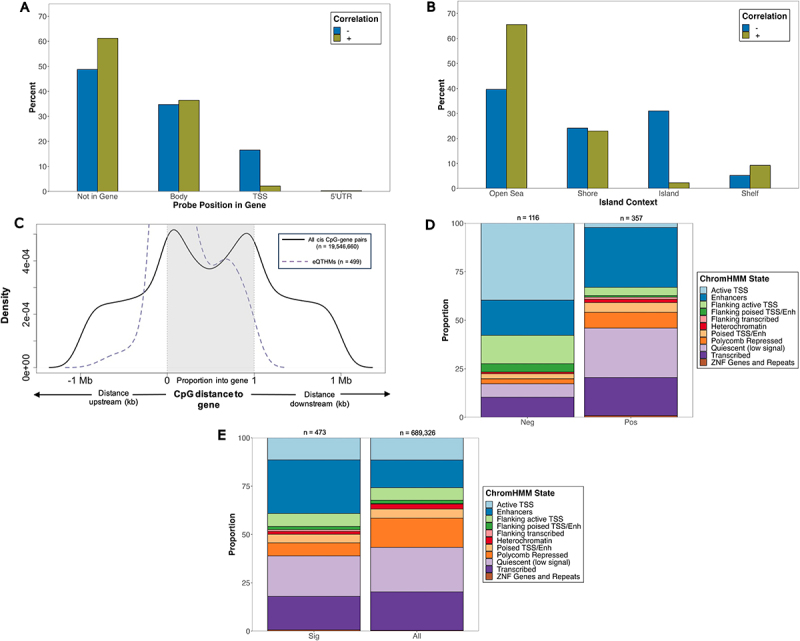
Characterization and distribution of genomic location of eQTHM signals (below empirical-p threshold, *n* = 499). (a) percentage of positively and negatively correlated eQthms across gene compartments of target gene. (b) percentage of positively and negatively correlated eQthms across CGI compartments. Fisher’s exact tests were used to test for enrichment of positively and negatively correlated eQthms across gene compartments (a) and CGI regions (b). (c) CpG distance from TSS/TTS of target eQTHM gene. TSS and TTS are represented by 0, 1 on x-axis, respectively. CpGs lying within gene have distance represented by proportion through gene. Shown are significant eQthms (dashed purple line) and all cis CpG-gene pairs from matrix eQTL (solid black line). (d,e) proportion of CpGs within ChromHMM states. Shown are negatively (Neg) and positively (Pos) associated CpGs (d), along with all significant CpGs (sig) and all CpGs on EPIC array (e). Fisher’s exact tests were used to test for enrichment of eQthms across ChromHMM states. Numbers on top of each bar represent # of CpGs in that group.

Among eQTHMs positively correlated with transcript abundance (positively correlated eQTHMs), we observed a depletion (OR = 0.60; 95% CI = 0.39, 0.93; *p* < 0.05) of CpGs that fall within their target gene (Figure 4(a)), along with an enrichment (OR = 2.89; 95% CI = 1.84, 4.57; *p* < 0.001) in ‘open sea’ regions of the genome (Figure 4(b)). Among eQTHMs negatively correlated with transcript abundance (negatively correlated eQTHMs), we observed an enrichment (OR = 8.93; 95% CI = 3.64, 24.16; *p* < 0.0001) in the TSS regions (Figure 4(a)) and in CGIs (OR = 19.45; 95% CI = 8.49, 50.44; *p* < 0.0001) (Figure 4(b)).

When plotting the distance between the eQTHMs and their associated gene, we observed a larger proportion of eQTHMs lying close to the TSS of the associated gene (Figure 4(c)). As the distance from the TSS increases in the upstream direction we observed a smaller proportion of eQTHMs in these areas. This pattern of increased proportions at the 5’ end of the genes and decreased proportions further away from the 5’ end was also observed across all cis eQTHM-gene pairs (Figure 4(c)).

We found a significant enrichment of eQTHMs among enhancers CpGs (OR = 2.29; 95% CI = 1.85, 2.80; *p* < 0.01) (Figure 4(e)), with particular enrichment of positively correlated eQTHMs in these regions (OR = 2.01; 95% CI = 1.17, 3.58; *p* < 0.01) (Figure 4(d)). Conversely, we observed an overall depletion of eQTHMs among CpGs in active TSS regions (Figure 4(e)), and among eQTHMs that did reside there, there was an overall enrichment of negatively correlated eQTHMs (OR = 28.36; 95% CI = 12.56, 72.85; *p* < 0.0001) (Figure 4(d)). Likewise, there was an overall depletion of eQTHMs in regions flanking active TSS (Figure 4(e)), though among eQTHMs in these regions, we observed an enrichment (OR = 3.90; 95% CI = 1.76, 8.72; *p* < 0.001) of negatively correlated eQTHMs (Figure 4(d)). We also found a strong enrichment of positively correlated eQTHMs in quiescent regions (OR = 4.61; 95% CI = 2.14, 11.37; *p* < 0.0001) (Figure 4(d)).

### DHMR analysis

We identified 107 significant (≥3 CpGs in DHMR and regional p-value <0.05) DHMRs across 100 genes (Table S2) and encompassing 479 unique CpGs. Among these CpGs, 85 (17.7%) were considered to have systematic 5hmC, representing a significant enrichment (OR = 2.96; 95% CI = 2.31, 3.75; *p* < 0.0001). Additionally, 121 of these CpGs (25.3%) were also eQTHMs.

**Figure 5.**
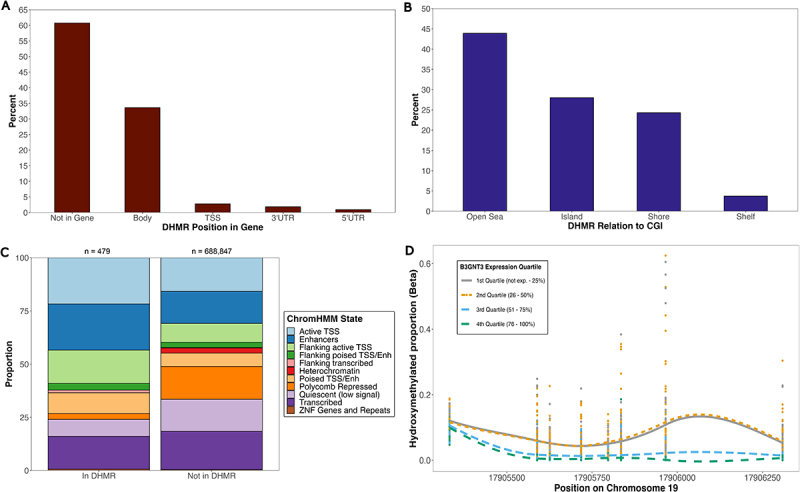
Characterization and distribution of transcription-associated DHMRs. Percentage of all significant DHMRs (*n* = 107) across (a) genic compartments and (b) CGI regions. (c) proportion of CpGs across ChromHMM states among CpGs in and out of DHMRs. Fisher’s exact tests were used to test for enrichment of CpGs across ChromHMM states. Numbers on top of each bar represent number of CpGs in that group. (d) most significant DHMR at B3GNT3 gene. Hydroxymethylated proportions among subjects at each of the 8 CpG probes (represented by vertical lines of stacked points) in the DHMR are shown. Data is stratified by B3GNT3 log_2_TMM expression quartiles among subjects. CpG site positions are displayed along the x-axis. Lines represent local regression model using the locally estimated scatterplot smoothing (LOESS) method.

Among the 107 significant DHMRs, 42 (39.3%) fell within their target gene, with most (*n* = 36, 33.6%) in the gene body (Figure 5(a)). Additionally, 47 DHMRs (43.9%) were in ‘open sea’ regions and 30 (28.0%) in CGIs (Figure 5(b)). We found an enrichment of DHMR CpGs compared to CpGs outside of DHMRs in active TSS regions (OR = 2.14; 95% CI = 1.71, 2.67; *p* < 0.0001), enhancers (OR = 1.65; 95% CI = 1.32, 2.06; *p* < 0.0001), regions flanking active TSS (OR = 2.69; 95% CI = 2.08, 3.45; *p* < 0.0001), and poised TSS or enhancers (OR = 2.19; 95% CI = 1.58, 2.96; *p* < 0.0001) (Figure 5(c)). We observed a depletion of DHMR CpGs in polycomb repressed regions (OR = 0.16; 95% CI = 0.08, 0.27; *p* < 0.0001) and quiescent regions (OR = 0.29; 95% CI = 0.20, 0.40; *p* < 0.0001) (Figure 5(c)).

The DHMR with the most significant association with expression is on chromosome 19 in the body of B3GNT3 (Figure 5(d)). This DHMR also overlaps with a poised TSS/enhancer region. We assessed the 5hmC proportion across the eight CpGs found in this DHMR, stratified by the B3GNT3 expression quartile across all subjects, and observed a negative correlation between 5hmC proportion and B3GNT3 transcript abundance across the DMHR (Figure 5(d)).

Interestingly, we found 29% of all DHMRs overlapped with an enhancer region. We also assessed whether DHMRs tend to be more associated with active transcripts than inactive transcripts, and found that the proportions were evenly distributed across transcript abundance quartiles (28%, 26.2%, 28%, and 17.8% of DHMRs were associated with transcripts in the first, second, third, and fourth quartile of transcript abundance, respectively). Among the 65 DHMRs that fell outside their target gene, 51.6% and 48.4% were located in the upstream and downstream direction, respectively.

## Discussion

In this study, we have achieved two distinct goals: 1) we have described the genomic landscape of hydroxymethylation in a healthy subset of infant placental tissue samples, and 2) we have identified the extent to which 5hmC is associated with placental gene expression. These findings will provide important reference data for future studies of 5hmC in placental tissue and offer insight into the roles this modification plays.

Although 5hmC proportions were relatively low across all placental samples, we found ~ 47,000 loci that met the criteria for demonstrating systematic placental hydroxymethylation (Figure 1(a)). Hydroxymethylation has been described as both an intermediate in the demethylation pathway [11], as well as a stable epigenetic mark [39]. By classifying 5hmC as systematic based on our criteria, we aim to distinguish 5hmC that fall within each of these categories. The criteria were established due to the notion that hydroxymethylation present as a result of cells undergoing active demethylation would likely be observed in only a limited number of samples, while stable, functional 5hmC regions would more likely be seen in a greater proportion of samples [19].

Our findings regarding the enrichment or depletion of systematic 5hmC at functionally relevant genomic regions (Figure 1(b)) are in strong concordance with results from previous placental studies. Green et al. [19] utilized placental methylation data from the 450 HumanMethylationBead Chip (450k) Array, and also observed a depletion of systematic 5hmC in areas like the TSS and particularly, CGIs. CGIs are often associated with promoters and have shown 5hmC depletion in brain tissue [40]. Similarly, Mora Hernandez et al. [17] profiled placental 5hmC using the 450k array, and in agreement with our results, found an enrichment of 5hmC in ‘open seas,’ along with a depletion in CGIs. Our 5hmC distribution results are also consistent with previous studies in brain samples; one study [41] utilized 450k data to quantify genome-wide patterns of 5hmC in the cerebellum and also observed significant depletion in CGIs, as well as enrichment in ‘shores,’ ‘shelves,’ and areas outside of CG-rich regions (‘open seas’). Another study by Spiers et al. [42] characterizing 5hmC and 5mC across human foetal brain tissue found about ¼ of the autosomal CpGs assayed were characterized by non-detectable 5hmC in all brain samples, with these sites enriched in CGIs and other regulatory regions including TSS. Among CpGs that contained detectable 5hmC, the authors noted these sites were enriched in ‘shores,’ ‘shelves,’ and gene bodies. Finally, this relationship has been replicated in a 5hmC study [43] of breast tissue, with the authors utilizing 450k methylation data to identify genomic loci containing elevated 5hmC. The authors observed 5hmC enrichment in ‘open seas’ and ‘shores,’ as well as a depletion in CGIs. Our 5hmC distribution findings, along with those from other studies of placental, brain, and breast tissue, further highlight the paradoxical relationship of 5hmC abundance in CpG-poor regions and depletion in CpG-rich regions. The strong concordance of systematic 5hmC in functionally relevant regions in placenta and other tissues sheds light on the potential shared regulatory pathways or processes involving 5hmC in these regions. Numerous mechanistic models have been proposed for how 5hmC is regulated in these areas, with one possible mechanism being that in CpG-rich regions, the CpGs are already methylated, and these methylated CpGs can recruit methyl-CpG binding domain (MBD) proteins to establish a constitutive heterochromatin state, thereby making TET1 inaccessible to the hypermethylated sites and preventing the conversion of 5mC to 5hmC [44]. Future studies of the placental 5hmC could examine specific chromatin marks and associated chromatin modifiers and binding proteins to understand these relationships.

Despite our 5hmC distribution results demonstrating agreement with previous findings in placenta, brain, and breast tissue, a portion of our findings are in contrast with those found in embryonic stem cells (ESCs). Specifically, studies in mouse ESCs have shown *increased* 5hmC at CGIs associated with bivalent (TET1/Polycomb repression complex 2 (PRC2)-cobound) promoters [45]. As TET1 is capable of binding to PRC2-repressed development regulators [46], it is possible that in mouse ESCs, TET1 may have a higher binding affinity to PRC2, thereby allowing for more oxidation of 5mC to 5hmC at CGIs in the bivalent promoters [46,47]. Additionally, mouse ESCs could have a greater proportion of CGIs bound by PRC2, in comparison to placental samples [48]. As TET1 binds to these areas, this in turn could manifest as an enrichment of 5hmC. As we observed a *depletion* of 5hmC in polycomb repressed regions (Figure 2), our findings are in direct contrast to those of ESCs. However, it is worth noting that our 5hmC distribution results are consistent with those from other differentiated cell types; a recent study [49] characterizing 5hmC in trophoblast stem cells (TSCs) within mouse placenta showed a profound lack of TET1 peaks overlapping with bivalent or polycomb repressed regions, leading to a subsequent reduction of 5hmC in these areas.

We observed that higher proportions of 5hmC, particularly in the gene body, are associated with more highly expressed genes (Figure 3). Previous studies have also shown 5hmC to be enriched in the gene body of active genes in mouse cerebellum [40] and that oxidation from 5mC to 5hmC prevents the binding of transcriptionally repressive MBD proteins [50]. Thus, it is possible that the observed enrichment of 5hmC in the gene body of actively transcribed genes could be due to the absence of MBD proteins in those areas, though future studies comprised of MBD-binding marks are needed to confirm this association.

Our study also sheds light on how 5hmC may be involved in regulating expression through regulatory elements, specifically enhancers. We observed an enrichment of 5hmC marks in enhancer regions (Figure 2), along with enrichment of eQTHMs in these regions (Figure 4(e)), with the majority of these eQTHMs being positively associated with expression (Figure 4(d)). Enrichment of 5hmC in these regions is in agreement with previous studies, which have shown that 5hmC tends to accumulate at poised and active enhancers labelled with H3K4me1, H3K18ac, and H3K27ac [51,52]. The trend we observed between 5hmC and expression in these regions could be due to the aforementioned increased variability of 5hmC in enhancer elements, and recent studies stress the importance of altered methylation patterns at enhancers as a critical component to variation in gene expression [53,54]. It is also possible that 5hmC at enhancers may be involved in changing the transcriptional landscape of placental tissue, which in turn aids in differentiation. The results from our DHMR analysis further support the notion that 5hmC may be regulating expression through enhancers; 29% of significant DHMRs overlapped with an enhancer region, as we observed an enrichment of DHMR CpGs in these regions (Figure 5(c)). As enhancers are known to regulate transcription of both distal and proximal genes [55,56], it is possible that 5hmC across contiguous CpGs in enhancers is working in concert to further promote transcription of the associated gene.

Our eQTHM findings are also noteworthy in that we observed an enrichment of systematic 5hmC among CpGs in eQTHMs (Table 2). This is especially significant given the small number of systematic 5hmC loci across all CpGs on the array (6.8% of all CpGs were deemed systematic). As these sites demonstrate consistently higher proportion of 5hmC *and* are significantly associated with gene expression, this provides a candidate list of loci with potentially functional 5hmC. Future studies should evaluate the functional relevance of these sites in the larger placental epigenome.

The most robust DHMR we identified was in the body of the *B3GNT3* gene (Figure 5(d)). Expression of *B3GNT3* is negatively correlated with 5hmC in the DHMR, meaning higher expression of *B3GNT3* associates with lower 5hmC proportions (Figure 5(d)). B3GNT3 is a transmembrane protein that has been found to be associated with immune cell infiltration and activation of the NF-kB pathway in gynaecologic cancers [57]. A previous study [58] looking at how mutations in IDH2 and TET2 cells modulates tumorigenesis in angioimmunoblastic T cell lymphoma (AITL) also found a negative correlation between CpG hypermethylation in a differentially methylated region (DMR) of *B3GNT3* and its corresponding expression levels.

To our knowledge, this study presents the most comprehensive description of the empirical relationship between placental 5hmC and gene expression through eQTHM analyses using a large sample size (*n* = 227) and the more comprehensive array (Illumina MethylationEpic array) than prior studies. Many of our findings are consistent with 5hmC-expression research in various other tissues and models [17,19,40,41,43]. We demonstrated that 5hmC is associated, for the most part positively, with gene expression in the placenta. Unlike previous placental epigenetic studies, we were able to leverage paired sample RNA-sequencing data to gain a better understanding of the relationship between 5hmC and expression. We were also very stringent in our control for multiple testing of the eQTHM analysis; by employing an empirical p-value threshold, we ensured that genes paired to a higher number of CpGs did not have a stronger chance of being a part of an eQTHM. We also utilized a DHMR analysis as an additional way to demonstrate the 5hmC and gene expression associations, which adds an additional level of rigour and confidence in the reported results. This study also benefits from the recruitment of a large cohort of placental samples; while previous placental epigenetics studies were generally conducted in a small cohort of samples, the RICHS cohort represents one of the largest cohorts of placental samples ever assembled, ensuring a well-powered study necessary to evaluate empirical effects. Finally, we employed a reference-based approach when estimating cell-type proportions in the placental samples, thereby limiting confounding due to inter-sample variability in cell compositions.

The results from this study are robust and relevant within the broader area of placental health, though these findings should be interpreted within the context of this study’s limitations. This is an observational study in which placental hydroxymethylation and expression were both measured in placenta at term. Therefore, we cannot conclude that our results are representative of the relationship between placental 5hmC and expression throughout development. Hydroxymethylation and expression being collected at the same time point also limits our ability to infer a causal relationship between the two. We also were unable to assess distal CpGs in our eQTHM analysis, mainly due to a limited sample size. Our DHMR analysis utilized eQTHM results with raw p-values <1 × 10^−4^, and thus there may be some false-positives in our DHMR hits. Finally, the RICHS cohort consists predominantly of healthy, white mothers and their infants from the New England region of the United States, and thus these findings may not be as generalizable to more diverse populations.

In summary, this study presents an important step in understanding the distribution and functional relevance of 5hmC in the placenta. Although additional studies are needed for a more complete understanding of placental hydroxymethylation, these findings serve as a good starting point for investigators looking to understand the role of 5hmC in placental epigenetics and add to the general body of evidence for a functional role of 5hmC.

## Supplementary Material

Table S2_All_Significant_DHMRs.csv

Table S1_All_Significant_eQTHMs.csv

Figure_S1_Prisma_Workflow.docx

## Data Availability

Raw data were generated at Emory University. Derived data supporting the findings of this study are available through the Gene Expression Omnibus (GEO, accession number: GSE144129).
