# Supplementary material for: Epigenetic landscape of 5-hydroxymethylcytosine and associations with gene expression in placenta
Source: Epigenetics. 2024 Mar 20;19(1):2326869. doi: 10.1080/15592294.2024.2326869 (PMC10956631; doi:10.1080/15592294.2024.2326869)
Supplement: Figure_S1_Prisma_Workflow.docx [file KEPI_A_2326869_SM9633.docx]

Placenta samples with available 5hmC data (n = 227)

Samples with available 5hmC ***and*** RNA-seq data (n = 197)

Samples excluded:

-Missing RNA-seq data (n = 30)

**Matrix eQTL Model**

(log_2_TMM transcript counts) = β_0_ + β_1_(5hmC M-values) + β_2_(Infant Sex) + β_3_(Birthweight Percentile) + β_4_(STB Proportions)

**Workflow of Main Analysis**

**5hmC**

**Distribution**

**eQTHM Analysis**

**DHMR Analysis**

**Empirical p-values**

1. Performed 1,000 permutations of Matrix eQTL model.
2. Modeled null (beta) distribution using permuted p-values for each gene.
3. Estimated gene-wise empirical p-values.
4. Calculated FDR for empirical p-values using Benjamini-Hochberg (BH) method.
5. Empirical-p threshold was defined as highest empirical-p among genes at FDR < 0.05 significance.
6. If nominal p-value for CpG was less than empirical-p threshold ***and*** gene had FDR < 0.05, it was called an eQTHM.

**Comb-p Package**

1. Input nominal Matrix eQTL p-values for each gene into comb-p.
2. Comb-p corrects p-values for auto-correlation with neighboring CpGs.
3. Used corrected p-value cutoff of 1e-4.
4. Comb-p uses specific cutoff to grow DHMRs for each gene and assign regional p-value to each.
5. DHMRs with regional p-value < 0.05 ***and*** ≥ 3 CpGs were called significant DHMRs.
